# Clinical malaria and the potential risk of anaemia among preschool-aged children: a population-based study of the 2015–2016 Malawi micronutrient survey

**DOI:** 10.1186/s40249-019-0607-8

**Published:** 2019-11-25

**Authors:** Peter Austin Morton Ntenda, Sosten Chilumpha, Edward Tisungane Mwenyenkulu, Jane Flora Kazambwe, Walaa El-Meidany

**Affiliations:** 1 Malaria Alert Centre, College of Medicine, University of Malawi, Private Bag 360, Chichiri, Blantyre 3 Malawi; 2 Ministry of Health and Population, Department of Planning and Policy Development, PO Box 30377, Lilongwe, Malawi; 3 The Malawi University of Science and Technology, P.O Box 5196, Limbe, Malawi; 4 NBS Bank PLC, Head Office NBS House Corner Chipembere Highway & Johnstone Roads Ginnery Corner Blantyre, P.O. Box 32251, Chichiri, Blantyre 3 Malawi; 5 Department of Nutrition, High Institute of Public Health, Alexandria University, Hiph 65 El-Horreya Avenue. El-Ibrahimia, Alexandria, Egypt

**Keywords:** Clinical malaria, Anaemia, α-Acid glycoprotein, Serum ferritin, Malawi

## Abstract

**Background:**

Anaemia and malaria are common and life-threatening diseases among preschool-aged children in many tropical and subtropical areas, and Malawi is no exception. Accordingly, this study aimed to examine the association of referral clinical malaria with anemia (hemoglobin [Hb] < 110 g/L) in preschool-aged children in Malawi.

**Methods:**

Using cross-sectional data obtained from the 2015–2016 Malawi Micronutrient Survey (MNS), multivariate logistic regression models were constructed using surveylogistic to account for the complex survey design. Blood samples of 1051 children aged 6–59 months were evaluated for malaria (using rapid diagnostic test [RDT] – SD BIOLINE Malaria Ag *P.f/*Pan test histidine-rich protein (HRP-II)™), Hb (using HemoCue 301), α-1-acid glycoprotein (AGP), and serum ferritin biomarkers (using simple sandwich enzyme-linked immunosorbent assay technique, ELISA) and inherited blood disorders from dry blood samples (DBS) using polymerize chain reaction (PCR). Diagnosis of clinical malaria was made on the basis of fever and a positive rapid diagnostic test (RDT).

**Results:**

Of the 1051 PSC analysed, 29% had anaemia while 24.4% had a referral to the hospital due to malaria. After adjustments for known confounders, PSC with a history of referral clinical malaria had increased odds of being anaemic (adjusted odds ratio [a*OR*] = 4.63, 95% confidence interval [*CI*]: 2.90–7.40), *P* <  0.0001.

**Conclusions:**

This study found that clinical malaria increased the risk of anaemia in PSC. Thus, elimination of malaria-causing parasites from the PSC’s blood should be rapid and complete in order to prevent the progression of uncomplicated malaria to a chronic infection that can lead to the development of malaria-related anaemia.

## Multilingual abstracts

Please see Additional file 1 for translations of the abstract into the five official working languages of the United Nations.

## Background

According to the World Health Organization (WHO), anaemia is a condition characterized by the reduced number of red blood cells (RBCs) and consequently their oxygen-carrying capacity is insufficient to meet the body’s physiologic needs [1]. Globally, anaemia is a public health problem with major consequences for human health as well as social and economic development [2]. Anaemia occurs at all stages of the life cycle but it is more pervasive in pregnant women and young children [3]. The global prevalence of anaemia is estimated at 24.8%, while the prevalence of anaemia in preschool-aged children is estimated at 47.4% [4]. In Malawi, according the 2015–2016 Malawi Demographic and Health Survey (MDHS), 63% of children suffers from some degree of anaemia [5]. Globally, approximately 50% of the cases of anaemia are due to iron deficiency (ID), although other conditions, such as folate, vitamin B12 and vitamin A deficiencies, chronic inflammation, parasitic infections such as malaria, and inherited disorders can all cause anaemia [6].

Malaria is also a major cause of morbidity and mortality among preschool-aged children in sub-Saharan Africa [7]. It is reported that 219 million new cases of malaria are reported annually, of which a big proportion is children under five years of age [8]. As of 2018, the WHO reported that 435 000 deaths owing to malaria had occurred worldwide, of which 403 000 deaths (approximately 93%) were recorded in the sub-Saharan Africa [9, 10]. Malaria is caused by *Plasmodium* parasites which are transmitted to people through the bites of infected female *Anopheles* mosquitoes [11]. In Malawi, through the Ministry of Health’s (MoH) National Malaria Control Program, the government has been scaling up the distribution of artemisinin-based combination therapies (ACTs), intermittent preventive treatment for pregnant women (IPTp) using sulfadoxine-pyrimethamine (SP), and insecticide-treated nets (ITNs) [12]. This development has led to a decline in under-five mortality from 234 deaths per 1000 live births in the 1990s to 63 deaths per 1000 live births in 2015 [5]. Unfortunately, despite this progress, malaria continues to be the leading cause of death among preschool-aged children in Malawi, accounting for 22% of all deaths of the under-five children. Furthermore, more than 50% of all admissions in Malawian hospitals among preschool-aged children are due to malaria [12]. Generally, malaria interventions are reported to reduce the risk of anaemia by 60% when using a diagnostic cut-off of 80 g/L [13].

Malaria parasites constitute one of the most complicated and multifactorial life cycle [14]. The *Plasmodium falciparum* involves an increased removal of parasitized and unparasitized RBCs through cytokine-mediated dyserythropoiesis and bone marrow suppression to iron delocalization [15]. Precisely, the malaria parasites invade the blood after an infective bite from female Anopheles mosquitoes and end up infecting the RBCs. At the end of that infection cycle, RBCs ruptures and releases more parasites into the bloodstream [16]. As such, this process reduces the number of RBCs resulting in moderate to severe anaemia. A great deal of intervention regarding malaria and anaemia in Malawi have been implemented [17–20] however little has been done to understand why the prevalence anaemia in preschool-aged children remains unacceptably high. Thus, the current study aimed to examine the likelihood of clinical malaria and the development of childhood anaemia in Malawi.

## Methods

### Study area

This study was conducted in Malawi, a sub-Saharan African country located south of the equator. Malawi is bordered by the United Republic of Tanzania, the People’s Republic of Mozambique, and the Republic of Zambia. Malawi has a tropical continental climate with maritime influences. Rainfall and temperature vary depending on altitude and proximity to the lake [21]. Malaria transmission is perennial in most areas and peaks during the rainy season from November to April. However, higher malaria transmission occurs along Lake Malawi and the lowland areas of the lower Shire Valley [12].

### Data source, and sampling method

**Fig. 1:**
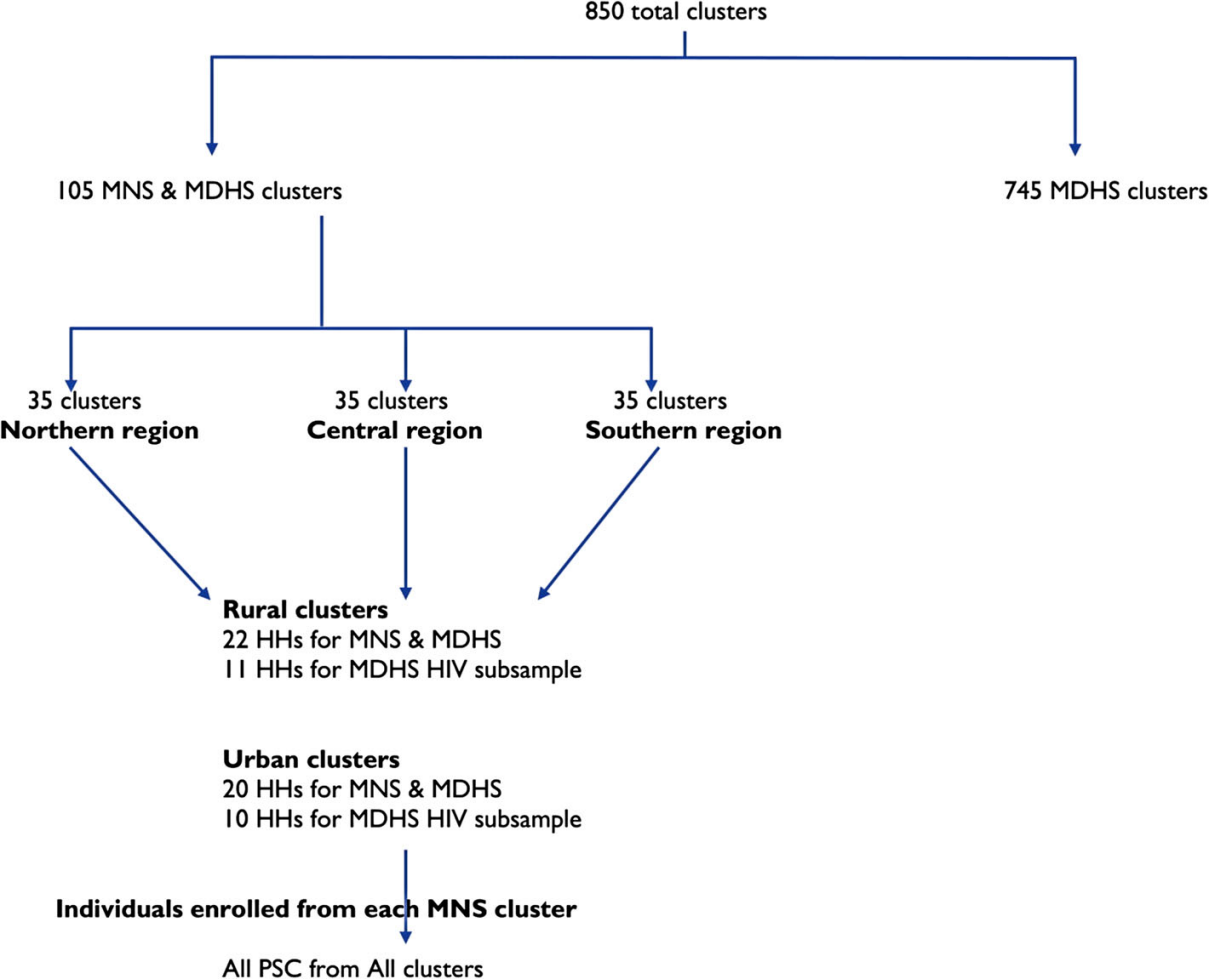
Allocation of the selected clusters and households with respect to region and residence

The current study utilized data taken from the 2015–2016 Malawi Micronutrient Survey (2015–16 MNS) [22]. The MNS was conducted jointly with the 2015–2016 MDHS between December 2015 and February 2016. The comprehensive methods used in this study can be obtained from the 2015–2016 MNS report. In brief, the 2015–2016 MDHS employed a two-stage sampling designed to produce a nationally representative sample. The first stage selected 850 clusters proportional to population. The second stage selected 27 516 households from the clusters with an equal probability systematic selection. The 2015–2016 MNS was selected as a subsample of the MDHS to produce estimates of the key indicators for the country as a whole and stratified by region and residence. A subsample of 105 clusters (35 clusters in each of the three regions) was randomly selected from the 850 MDHS clusters. Figure 1 shows the allocation of selected clusters and households with respect to residence and region.

### Data collection and study sample

Data were collected from women aged 15–49 with children younger than five years of age prior to the survey using standard questionnaires. In the DHS data, of the 25 146 eligible households, 24 562 were successfully interviewed representing a 98% response rate. Information on sociodemographic, household and economic factors were collected through face-to-face interviews. As per WHO recommendations, analysis of the current study was restricted to all PSC aged 6–59 months [1].

### Field and laboratory procedures

An axillary temperature of every child was documented where by fever was defined as an axillary temperature of equal to or greater than 37.5 °C. Regarding to laboratory, approximately 7 ml of blood samples were collected from PSC for biochemical and haematological tests. About 5 ml of the blood sample was transferred into trace free elements (Royal Blue Top) test tube and 2 ml into Purple Top – Ethylenediaminetetraacetic acid (EDTA) test tube [22]. About 10 μl of whole the blood sample from EDTA vacutainer was used to tested anaemia (Hb concentration) using HemoCue® 301 system (Ängelholm, Sweden) and another 10 μl for malaria using a rapid diagnostic test (RDT) kit the Standard Diagnostic (SD) BIOLINE Malaria Ag *P.f*/Pan histamine-rich protein (HRP-II)™ RDT) – Standard Diagnostics Inc., Suwon City, South Korea [23, 24]. The remaining blood from EDTA vacutainer was used to test for RBC folate in women of reproductive age and inherited blood disorders (alpha thalassemia, G6PD, and sickle cell using PCR) in PSC. The plasma from EDTA vacutainer was store at − 20 °C as back in CHSU laboratory. On the other hand, 100 μL of serum from Royal Blue Top was transferred into polymerize chain reaction (PCR) vials and were ship to German for the biochemical examination of Ferritin and Alpha-1-Acid Glycoprotein (AGP) along with other parameters such as Soluble Transferrin Receptor (sTfR), C-reactive protein (CRP), and Retinol Binding Protein (RBP) [22]. A combined measurement of the biochemical tests for ferritin and AGP and other biochemical micronutrient parameters were performed using an inexpensive, sensitive, and simple sandwich enzyme-linked immunosorbent assay (ELISA) technique in the VitMin laboratories in German [25]. To identify the glucose-6-phosphate dehydrogenase (G6PD) deficiency, the widely known G6PD A — variant was investigated using three commercially available TaqMan probe sets (Applied Biosystems Foster City, CA, USA); A376G (rs1050829) distinguished A and B isoforms, G202A (rs1050828) identified the A — variant, and sex was scored using SRY_VIC and ABCD1_CCHS0H-FAM for Y and X chromosomes, respectively. Each assay included positive and negative controls, with random sample duplicates [6]. To detect alpha-thalassemia trait, the 3.7-kb α-globin gene deletion (−α^3.7^) was rated using a copy-number variant TaqMan assay with custom TaqMan probes, as described elsewhere [26]. The procedure for the identification of sickle status has been described before [6]. In brief, amplification of the β-globin gene was performed using forward (5′-TGC TTA CCA AGC TGT GAT TCC-3′) and reverse (5′-CTT CCT ATG ACA TGA ACT TAA CCA-3′) primers. Haemoglobin S (HbS) mutation (Glu6Val) was evaluated by a custom TaqMan probe (RT-PCR) designed for the HbS (sickle, rs334) polymorphism.

### Measures

#### Dependent variable

Childhood anaemia was the dependent variable of this study. Using the WHO recommendations, anaemia in PSC was characterized as children with Hb concentration < 110 g/L after adjusting for altitude [1]. Altitude-adjusted Hb concentration less than 40 g/L or greater than 180 g/L were considered extreme and excluded from the analysis [27].

#### Main independent variable

Clinical malaria was the main independent variable assessed in this study. Clinical malaria was defined as any PSC with malaria-related symptoms such as fever – an axillary temperature ≥ 37.5 °C, chills, severe malaise, headache or vomiting at the time of examination or 48 h prior to the examination in the presence of a *Plasmodium falciparum* positive blood smear [28]. However, in this study, a positive *Plasmodium falciparum* result was confirmed using the RDT [22].

#### Covariates

The covariates included in this study were; age of the child in months, sex of the child, and fever in last two weeks, malaria test result, α-1-acid glycoprotein (AGP), serum ferritin, α-thalassemia, household hunger scale, type of place of residence, and region of residence were used as covariates. The sex was grouped as (male/female), the age of the children was categorized as 6–11, 12–23, 24–35, 36–47, and 48–59 months. As regards fever in the last two weeks, the respondents were asked if the child had a fever (yes/no) in the last two weeks, while malaria test result was categorized as positive or negative. AGP and serum ferritin were grouped as normal or abnormal. Abnormal value for AGP was AGP levels of greater than 1 g/L and abnormal levels for serum ferritin were less than 12 μg/L. Serum ferritin adjusted for inflammation using internal regression approach [29]. The household hunger scale was categorized as little to no hunger and moderate to severe hunger using the recommendations from the Food and Nutrition Technical Assistance III Project (FANTA) [30]. The type of place of residence (rural and urban), and region of residence (northern, central, and southern) were used to assessed area of residence and region respectively.

### Statistical analysis

All statistical analyses were conducted using SAS software version 9.4 (SAS Institute, Cary, NC, USA). To account for the complex survey design, the survey-specific SAS procedures for weighting, clustering, and stratification in the survey design (SurveyFreq) was used to report the basic statistics. Baseline characteristics were reported as weighted frequency and percentages. The *P*-values from the bivariate analyses were reported using Rao-Scott Chi-Square to test the differences between groups anaemic (yes/no). All factors that showed significance at *P* ≤ 0.25 in the bivariable analyses were fitted into the final models of the regression analyses in order to have the best fit statistical model. The multivariate regression analyses were performed using Surveylogistic where anaemia was the dependent variable and characteristics such as sociodemographic, clinical, biochemical, and inherited blood disorders were the independent variables. Adjusted odds ratio (a*OR*) with their *p*-values and 95% confidence interval (*CI*) were reported.

### Ethical statement

Protocols for the MNS 2016 were approved by the National Health Sciences Research Committee (NHSRC) and the Institutional Review Board (IRB) of ICF Macro (https://dhsprogram.com/What-We-Do/Protecting-the-Privacy-of-DHS-Survey-Respondents.cfm). The survey was implemented by the National Statistics Office (NSO) and the Community Health Sciences Unit (CHSU). At the beginning of each interview and prior to blood collection, informed consent from MNS eligible households and the survey participants were sought. Furthermore, community leaders provided the consent of survey activities. ICF Macro IRB ensures that the survey is in line with the U.S. Department of Health and Human Services regulations for the protection of human subjects (45 CFR 46), while the NHSRC ensures that the survey was conducted with laws and norms of the Malawi.

## Results

### Baseline characteristics of the study participants

Of the 1051 PSC, 29.0% had anaemia (Hb < 110 g/L). The prevalence of clinical malaria was 27.4%. Similarly, the prevalence of *Plasmodium falciparum* positive RDT was 27.6%. The prevalence of fever in the last two weeks was 43.3%. Table 1 reports the baseline characteristics of the study sample. Approximately, 51% of the children were male and a majority of children (56%) had abnormal levels of α-1-acid glycoprotein while about 22% had abnormal levels of serum ferritin. Furthermore, a majority of children did not have G6PD (72%) nor α-thalassemia (60%). A majority of children (60%) were residing in household with moderate to severe hunger and more than three-quarter (89%) of the children were rural dwellers.

**Table 1:** Sociodemographic, comorbidities and biochemical characteristics of the study sample

Variable	Frequency *n* = 1051	Percent (%)
Sex		
Female	539	49.18
Male	512	50.82
Age (months)		
6–11	97	9.43
12–23	221	21.77
24–35	257	25.29
36–47	264	24.00
48–59	212	19.52
Fever in last 2 weeks		
Yes	505	43.25
No	546	56.75
Malaria test result^†^		
Positive	278	27.63
Negative	773	72.37
Clinical malaria^a^		
Yes	271	27.37
No	780	72.63
Alpha1-Acid Glycoprotein		
Normal	443	44.33
Abnormal	608	55.67
Serum ferritin^b^		
Normal	835	78.29
Abnormal	216	21.71
G6PD (*n* = 1004)		
Unaffected	723	72.08
Affected	118	11.76
Carrier	162	16.15
Alpha-thalassemia (*n* = 999)		
Unaffected	609	60.96
Affected	80	8.01
Carrier	310	31.03
Household hunger scale		
Little to none	485	39.98
Moderate to severe	566	60.02
Type of place of residence		
Urban	117	10.03
Rural	934	89.97
Region of residence		
North	372	13.82
Central	383	44.34
Southern	296	41.84
*Outcome variable*		
Any anemia		
< 110 g/L	742	70.96
≥ 110 g/L	309	29.04

*G6PD* Glucose-6-phosphate dehydrogenase

^a^Defined as an individual with malaria-related symptoms (fever [axillary temperature ≥ 37.5 °C], chills, severe malaise, headache or vomiting) at the time of examination or 1–2 days prior to the examination in the presence of a *Plasmodium falciparum* positive blood smear; ^b^Serum ferritin adjusted for inflammation using internal regression approach, ^†^malaria test using a rapid diagnostic test (RDT) kit the Standard Diagnostic (SD) BIOLINE Malaria Ag P.f/Pan histamine-rich protein (HRP-II)™ RDT)

### Results of the bivariate analysis

**Fig. 2:**
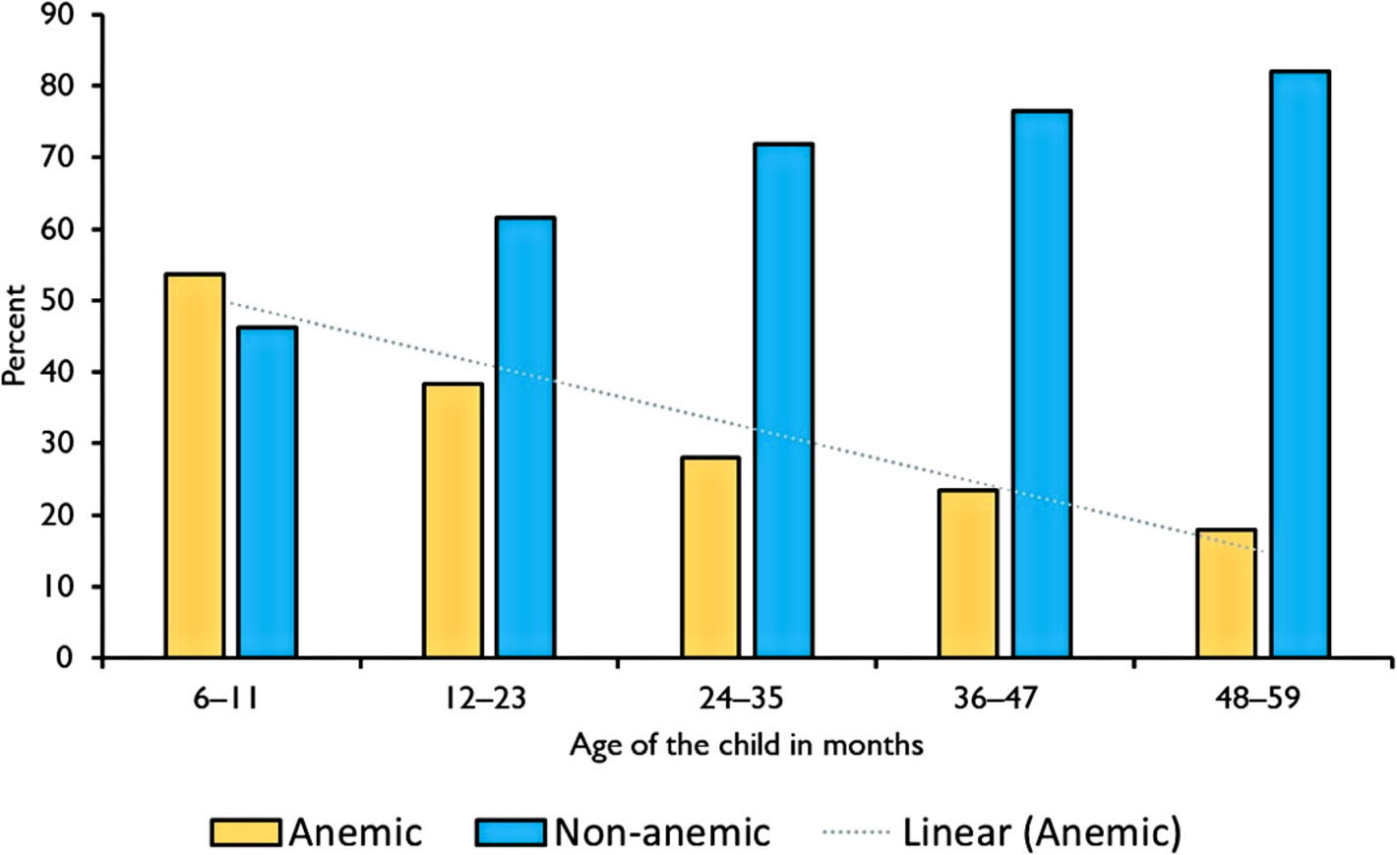
Prevalence of anaemia by the age of the child

Table 2 presents the prevalence of anaemia by clinical and demographic characteristics of study sample. The prevalence of anaemia was significantly high in PSC of age group 6–11 months (*P* <  0.0001) – Fig. 2 presents the prevalence of anaemia by age of the child. The prevalence of anaemia was also high in children with a history of fever in the last two weeks (*P* <  0.0001), in PSC with a positive RDT (*P* <  0.0001), in PSC with referral clinical malaria (*P* <  0.0001). Furthermore, the prevalence of anaemia was high in PSC with abnormal levels of AGP (*P* <  0.0001) and serum ferritin (*P* <  0.0001).

**Table 2:** Sociodemographic, comorbidities and biochemical characteristics by anemia^a^

Variable	Non-anemic *n* (%)	Anemic^a^ *n* (%)	*P* value
Sex			0.5618
Female	374 (69.39)	165 (30.61)	
Male	368 (71.88)	144 (28.13)	
Age (months)			< 0.0001
6–11	45 (46.39)	52 (53.61)	
12–23	136 (61.54)	85 (38.46)	
24–35	185 (71.98)	72 (28.02)	
36–47	202 (76.52)	62 (23.48)	
48–59	174 (82.08)	38 (17.92)	
Fever in last 2 weeks			< 0.0001
Yes	320 (63.37)	185 (36.63)	
No	422 (77.29)	124 (22.71)	
Malaria test result^†^			< 0.0001
Positive	127 (45.68)	151 (54.32)	
Negative	615 (79.56)	158 (20.44)	
Clinical malaria^b^			< 0.0001
Yes	123 (45.39)	148 (54.61)	
No	619 (79.36)	161 (20.64)	
Alpha1-Acid Glycoprotein			< 0.0001
Normal	366 (82.62)	77 (17.38)	
Abnormal	376 (61.84)	232 (38.16)	
Serum ferritin^c^			< 0.0001
Normal	613 (73.41)	222 (26.59)	
Abnormal	129 (59.72)	87 (40.28)	
G6PD			3.0684
Unaffected	514 (71.09)	209 (28.91)	
Affected	82 (68.91)	37 (31.09)	
Carrier	116 (71.60)	46 (28.40)	
Alpha-thalassemia			< 0.0001
Unaffected	445 (73.07)	164 (26.93)	
Affected	39 (48.75)	41 (51.25)	
Carrier	223 (71.94)	87 (28.06)	
Household hunger scale			0.3706
Little to none	349 (71.96)	136 (28.04)	
Moderate to severe	393 (69.43)	173 (30.57)	
Type of place of residence			0.1684
Urban	89 (76.07)	28 (23.93)	
Rural	653 (69.91)	281 (30.09)	
Region of residence			0.6143
North	257 (69.09)	115 (30.91)	
Central	277 (72.32)	106 (27.68)	
Southern	208 (70.27)	88 (29.73)	

*G6PD* Glucose-6-phosphate dehydrogenase

^a^Hb < 110 g/L; ^b^defined as an individual with malaria-related symptoms (fever [axillary temperature ≥ 37.5 °C], chills, severe malaise, headache or vomiting) at the time of examination or 1–2 days prior to the examination in the presence of a *Plasmodium falciparum* positive blood smear; ^c^Serum ferritin adjusted for inflammation using internal regression approach, ^†^malaria test using a rapid diagnostic test (RDT) kit the Standard Diagnostic (SD) BIOLINE Malaria Ag P.f/Pan histamine-rich protein (HRP-II)™ RDT)

### Multivariable results of clinical malaria and anaemia

Table 3 shows unadjusted and adjusted odds ratio of clinical malaria on anemia. PSC who had a history of referral clinical malaria, were over four times (a*OR* = 4.63; 95 *CI*: 2.90–7.40); *P* <  0.0001, compared to those PSC without a history of clinical malaria even after adjusting for known factors associated with anaemia. Additionally, the odds of anaemia were high in PSC of the age group 6–11 months (a*OR* = 4.40; 95% *CI*: 1.82–10.61); *P* = 0.0003, in PSC with a history of fever in the last two weeks (a*OR* = 1.58; 95% *CI*: 1.04–2.39); *P* = 0.0324, in children with high levels of AGP (a*OR* = 2.13; 95% *CI*: 1.36–3.34); *P* = 0.0010, those with iron deficiency (a*OR* = 2.37; 95% *CI*: 1.40–4.02); *P* = 0.0014, and those with alpha-thalassemia disease (a*OR* = 4.08; 95% *CI*: 1.74–9.59); *P* = 0.0004.

**Table 3:** Association of clinical malaria and anemia^a^ in preschool-aged children

Variable	cr*OR* (*CI*)		*P* value	a*OR* (*CI*)		*P* value
Clinical malaria^b^						
Yes	3.78	(2.55–5.59)	< 0.0001	4.63	(2.90–7.40)	< 0.0001
No	1.00			1.00		
Age (months)						
6–11	4.87	(2.39–9.93)	< 0.0001	4.40	(1.82–10.61)	0.0003
12–23	2.66	(1.46–4.86)	0.0399	2.18	(1.05–4.56)	0.1179
24–35	1.29	(0.73–2.30)	0.0450	0.90	(0.44–1.88)	0.0071
36–47	1.20	(0.67–2.16)	0.0186	1.11	(0.56–2.21)	0.0733
48–59	1.00			1.00		
Fever in last 2 weeks						
Yes	1.75	(1.21–2.53)	0.0029	1.58	(1.04–2.39)	0.0324
No	1.00			1.00		
Alpha1-Acid Glycoprotein						
Abnormal	2.50	(1.64–3.81)	< 0.0001	2.13	(1.36–3.34)	0.0010
Normal	1.00			1.00		
Serum ferritin^c^						
Abnormal	1.97	(1.26–3.09)	0.0031	2.37	(1.40–4.02)	0.0014
Normal	1.00			1.00		
Alpha-thalassemia						
Unaffected	1.01	(0.66–1.53)	0.0030	0.90	(0.57–1.42)	0.0018
Affected	3.62	(1.84–7.10)	< 0.0001	4.08	(1.74–9.59)	0.0004
Carrier	1.00			1.00		
Type of place of residence						
Urban	1.27	(0.53–3.01)	0.5981	2.17	(0.97–4.89)	0.0601
Rural	1.00			1.00		

cr*OR*: Crude odds ratio; a*OR*: Adjusted odds ratio; *CI*: Confidential interval

^a^Anemia defined as Hb < 110 g/L; ^b^defined as an individual with malaria-related symptoms (fever [axillary temperature ≥ 37.5 °C], chills, severe malaise, headache or vomiting) at the time of examination or 1–2 days prior to the examination in the presence of a *Plasmodium falciparum* positive blood smear; ^c^Serum ferritin adjusted for inflammation using internal regression approach

## Discussion

The aim of this study was to examine the likelihood of clinical malaria and the development of childhood anaemia in Malawi. This is the first study to report the association of clinical malaria and the likelihood of anaemia in PSC using a nationally representative sample in Malawi. Accordingly, this study revealed that children who had clinical malaria were more likely to develop anaemia.

**Fig. 3:**
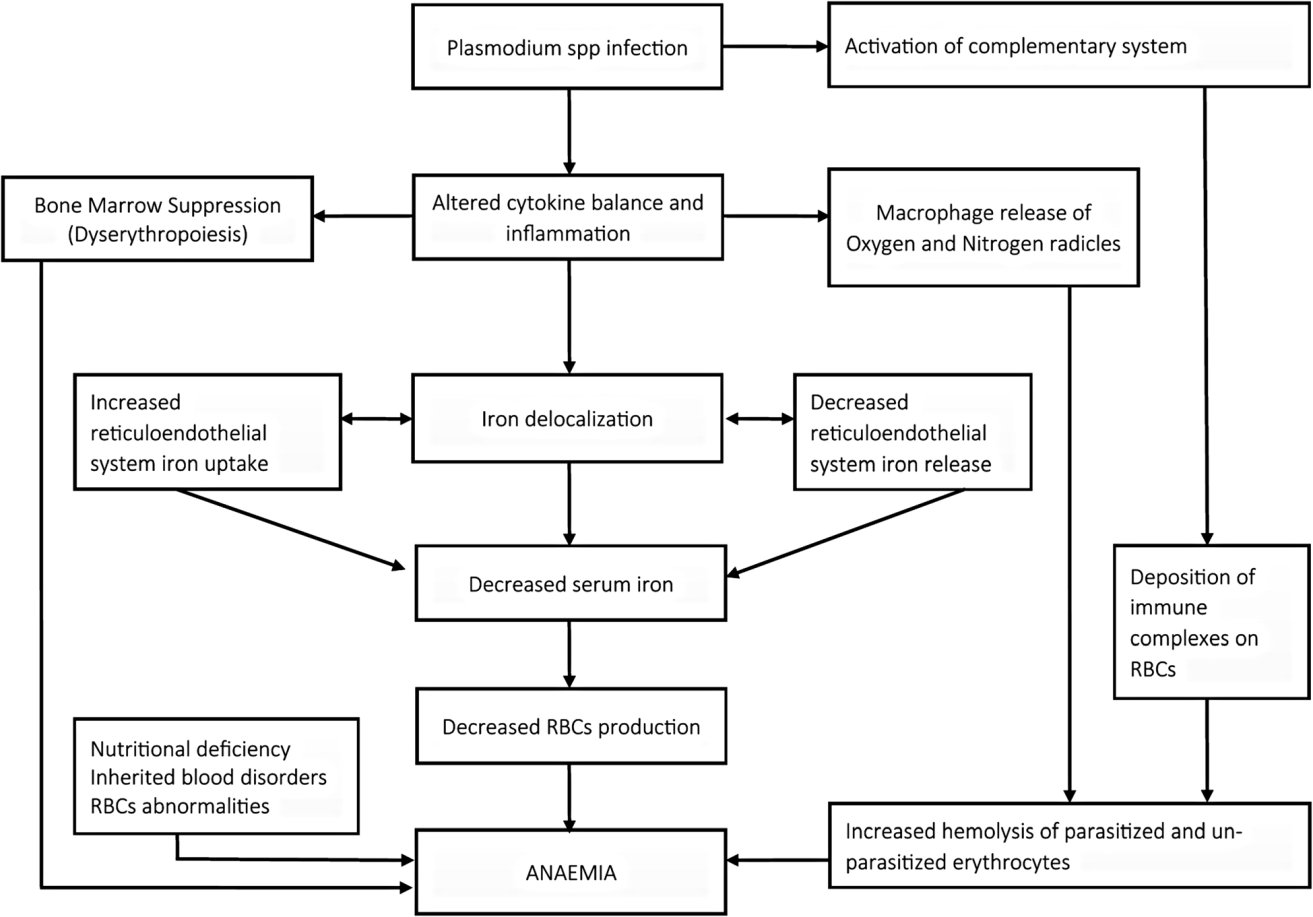
The pathogenesis of malaria infection and anemia (Adapted from C.V. Nweneka et al., 2010) [33]. RBC: Red blood cells

Anaemia occurs when RBCs are destroyed at an increased rate than the rate at which are supposed to be replaced or when RBC production falls below the rate at which the body requires to maintain a steady state [31]. The pathogenesis of malarial-anaemia is said to be multifactorial but the exact mechanisms behind several haematology changes in the course of malaria is poorly understood [32]. However, this process involves a great deal of phenomenon including immune and non-immune mediated destruction of the parasitized and non-parasitized RBCs (pRBC’s and npRBC’s), bone marrow impairment, altered cytokine balance, nutritional deficiency, and interactions with common hemoglobinopathies and erythrocyte defects and thalassemia which compromises rapid recovery from anaemia – Fig. 3 presence the pathogenesis of malaria infection and anemia [33, 34].

Malarial infection begins when sporozoites are inoculated together with anti-coagulant saliva in the course of a blood meal of an infected *Anopheles* mosquito [16, 35, 36]. Following inoculation of the sporozoites, the parasites travel to the liver in search of a conducive environment in the hepatocyte and successfully replicate in the liver, after which newly formed merozoites are released back in the bloodstream [16, 37]. During the blood stage of the asexual developmental cycle, malaria parasites replicate inside RBCs. As this process continues more RBCs get destroyed and merozoites induce changes in RBC membranes and increase splenic activities hence increase destruction and clearance of parasitized RBCs [38].

Infection with *Plasmodium* species induce homeostatic imbalance and lower Hb concentration thus resulting in anemia [34]. It has been reported that imbalance of cytokines such as tumor necrosis factor alpha (TNF-α), interleukin-6 (IL-6), IL-10 and interferon gamma (IFN-γ) resulting from malaria related-inflammation can induce changes in iron absorption and distribution (iron delocalization) [39]. Iron delocalization end up in decreasing the release of iron from the reticuloendothelial system as well as increasing uptake of iron from the reticuloendothelial system thus resulting in iron accumulation in tissues/secretions and iron deficiency in blood [39, 40]. Generally, hepcidin, an iron regulatory hormone is a crucial determinant in the relationship between inflammation and anaemia. It is known that pro-inflammatory cytokines secretion is up-regulated by hepcidin from macrophages and hepatocytes which in turn inhibits iron absorption and its release from macrophages by down-regulating the concentration of ferroportin thereby contributing to iron deficiency anaemia through the reduction of RBCs production [41].

Another pathway by which an altered cytokines balance and inflammation can induce anemia is through bone marrow depression (dyserythropoiesis) and erythrophagocytosis following low IL-10 and IL-12 or excess of T helper cell type 1 (th1), cytokines follicular helper T cells (TFH), TNF- α and nitric acid (NO) [42]. Often times, dyserythropoiesis induces the morphological abnormalities of the erythroid series which include multinuclearity of the normoblasts, intracytoplasmic bridging, karyorrhexis, incomplete and unequal mitotic nuclear divisions in RBCs of the individuals infected with malaria [43–45], hence induces erythrophagocytosis of the parasitized and unparasitized RBCs activities. Additionally, the mechanism that leads to reduced production of RBCs may be due to erythroid hypoplasia induced by inflammation [46]. The normal response to erythropoietin is suppressed due to an autologous serum factor. This may further suppress the growth of early precursors of RBCs including the burst forming unit- erythroid (BFU-E) and colony forming-unit erythroid (CFU-E) [47, 48]. Furthermore, another pathway by which malarial infection increases erythrophagocytosis of the parasitized and unparasitized RBCs is through activation of complement and deposition of immune complexes on RBCs by cytotoxic natural killer and natural killer cells [49–51]. It has been reported that complement plays a role in the occurrence of anemia in the course of malaria infection by opsonization of unparasitized RBCs with C3 fragments which in turn can lead to phagocytosis of RBCs [52, 53].

In addition to clinical malaria, the other characteristics such as the age of the child, a history of fever in the last two weeks, AGP, serum ferritin, and alpha thalassemia were significantly associated with anaemia in PSC. The results of age are in line with the past research where it was reported that anaemia is frequent among children around the time of the growth spurt, especially between the ages of 6 and 24 months [54, 55]. During the infant growth spurt (6–12 months), the physical development is rapid, and the blood volume is largely expanded, whereas the iron storage from the maternal source has usually been depleted. Therefore, an inadequate intake of exogenous iron during this period could lead to anaemia [56]. Fever is a common response that occurs as the result of infection and inflammatory diseases and it goes beyond the site of infection [57]. It is known that some pathogens can end up in the production of pyrogens, chemicals that efficiently modify the thermostat status of the hypothalamus to raise body temperature and cause fever [58]. Generally, pyrogens may induce the leukocytes to release endogenous pyrogens such as interleukin-1 (IL-1), IL-6, interferon-γ (IFN-γ), and tumour necrosis factor (TNF) [58, 59]. In turn, these molecules can then trigger the release of prostaglandin E2 (PGE2) from other cells, resetting the hypothalamus to initiate fever [58, 59]. We also found that children with alpha-thalassemia had increased odds of being anaemic. Alpha-thalassemia is inherited as an autosomal recessive disorder which is characterized by a microcytic hypochromic mild anaemia and a clinical phenotype varying from almost asymptomatic to lethal haemolytic anaemia [60]. Furthermore, children with abnormal levels of serum ferritin and AGP levels were significantly more likely to be anaemic. AGP is a measure of chronical inflammation while serum ferritin is a measure of iron deficiency. The mechanisms through which both of these parameters result in anaemia have been explained earlier in this paper and elsewhere [61].

### Strengths and limitations

The findings can be generalized in the Malawian context due to the use of the nationally representative sample. The use of malaria RDTs was helpful since they can assist in making a rapid, accurate diagnosis in circumstances where the microscopy-based diagnosis may be unreliable or available. The use of serum ferritin and alpha-thalassemia makes our results on clinical malaria to be robust and valid. However, the use of cross-sectional study design cannot provide a causal relationship between the explanatory variable and anaemia. The HemoCue system was used for assessment of Hb levels. Additional studies may need the use of other Hb indices to define anaemia. The RDT may not be able to detect some infections with lower numbers of malaria parasites circulating in the patient’s bloodstream. In addition, all positive RDTs should be followed by microscopy.

## Conclusion

In this study we aimed to examine the likelihood of clinical malaria and the development of anaemia among PSC in Malawi. Indeed, the results of this study have demonstrated that clinical malaria is a potential risk factor for anaemia in PSC. Thus, clearance of the *Plasmodium* parasite from the PSC’s blood should be rapid and complete in order to prevent progression of uncomplicated malaria to a chronic infection that leads to malaria-related anaemia. The results of this study have some policy implications, i.e. programmes that aim at combating anaemia in infants and young children should not focus on iron supplementation, deworming treatment, and complementary feeding practices as strategies to manage anemia, diagnosis and case management of malaria should also be considered promptly as it has been proved by this study that clinical malaria is associated with anaemia in PSC thus, posing threats to the strategies that were out in place to tackle anaemia in Malawi.

## Supplementary information

**Additional file 1:** Multilingual abstracts in the five official working languages of the United Nations.

## Data Availability

The datasets generated and/or analysed during the current study are available in the MEASURE DHS repository; https://dhsprogram.com/data/

## Abbreviations

AGP: Alpha-1-acid glycoprotein
AOR: Adjusted odds ratio
CDC: Center for Disease Control
CFR: Code of Federal Regulations
CHSU: Community Health Sciences Unit
*CI*: Confidence interval
CRP: C-reactive protein
EDTA: Ethylenediaminetetraacetic acid
ELISA: Enzyme-linked immunosorbent assay
Fer: Ferritin
g/dL: grams per deciliter
Hb: Hemoglobin
HIV: Human immunodeficiency virus
ID: Iron deficiency
IL: Interleukin
INF: Interferon
IRB: Institutional Review Board
MDHS: Malawi Demographic and Health Survey
MNS: Malawi Micronutrient Survey
MPHC: Malawi Population and Housing Census
NHSRC: National Health Sciences Research Committee
NC: New York
NSO: National Statistical Office
a*OR*: Adjusted odds ratio
PCR: Polymerase chain reaction
PGE: Prostaglandin E
PSC: Preschool children
RBCs: Red blood cells
RBP: Retinol Binding Protein
RDTs: Rapid Diagnostic Test
SAS: Statistical analysis software
SEAs: Enumeration areas
sTfR: Soluble Transferrin Receptor
TNF: Tumor necrosis factor
USA: United States of America
VIF: Inflation factor
WHO: World Health Organization
