# Supplementary material for: Clinical malaria and the potential risk of anaemia among preschool-aged children: a population-based study of the 2015–2016 Malawi micronutrient survey
Source: Infect Dis Poverty. 2019 Nov 25;8:95. doi: 10.1186/s40249-019-0607-8 (PMC6876103; doi:10.1186/s40249-019-0607-8)
Supplement: Supplementary file 1 — Additional file 1: Multilingual abstracts in the five official working languages of the United Nations. [file 40249_2019_607_MOESM1_ESM.pdf]

Translation of the abstract into the five official working languages of the United Nations

الإصابة بالملاريا والمخاطر المحتملة للإصابة بمرض فقر الدم بين الأطفال في سن ما قبل المدرسة: دراسة استقصائية للمغذيات الدقيقة أجريت على سكان دولة مالاوي عام 2015-2016

بيتر أوستن مورتون نتندا, سوستين تشيلومفا ، إدوارد تيسونجان موينينكولو ، جين فلورا كازامبوي ، ولاء الميداني

ملخص

المعلومات الأساسية: يعتبر مرضا الملاريا وفقر الدم من الأمراض الشائعة واللذان يهددان حياة الأطفال في سن ما قبل الدراسة في العديد من المناطق الاستوائية وشبه الاستوائية ومن ضمنها دولة مالاوي. ووفقًا إلى ذلك، تهدف هذه الدراسة إلى دراسة ارتباط الإصابة بمرض الملاريا بعدد الحالات المُحالة للطبيب بسبب فقر الدم (أي أن نسبة الهيموغلوبين لديهم تكون أقل من 110 جرام / لتر) في الأطفال في سن ما قبل المدرسة في مالاوي.

منهجية الدراسة: البيانات الناتجة من الدراسة الاستقصائية للمغذيات الدقيقة بالطريقة المستعرضة (MNS) التي أُجريت في 2015-2016 استخدمت في تصميم نماذج للانحدار اللوجستي متعدد المتغيرات بمساعدة المسح الإحصائي وذلك لحساب تصميم دراسة استقصائية معقدة. فحصت عينات دم 1051 طفل تتراوح أعمارهم بين 6 إلى 59 شهرًا لتقييم إصابتهم بالملاريا عن طريق الاختبارات المعملية الآتية: اختبار التشخيص السريع [RDT] وهو -SD BIOLINE Malaria AgP.f/ Pan test histidine-rich protein (HRP-II)™، ونسبة الهموجلوبين في الدم باستخدام HemoCue 301، واختبار ألفا - 1- جليكوبروتين الحمضي (AGP)، واختبار المؤشرات الحيوية للفيريتين بمصل الدم باستخدام المقايسة الامتصاصية المناعية للإنزيم المرتبط ، الإليزا، واختبارات أمراض الدم الوراثية من عينات الدم الجافة (DBS) باستخدام تفاعل البوليميراز المتسلسل (PCR). تم تشخيص الملاريا على أساس الحمى واختبار التشخيص السريع الإيجابي (RDT).

النتائج: بعد تحليل نتائج الـ1051 طفل في سن ما قبل الدراسة ، كانت نسبة الأطفال المصابين بفقر الدم 29% بينما كانت نسبة الأطفال المُحالين إلى المستشفى نتيجة إصابتهم بالملاريا 24.4%. بعد إجراء التعديلات على المتغيرات المشوشة المعروفة، زادت نسبة الأطفال في سن ما قبل الدراسة المصابين بفقر الدم والذين لديهم تاريخ مرضي مسبق بالإصابة بالملاريا (نسبة الأرجحية المعدلة [الـOR] تساوي 4.63، ومجال الثقة لـ 95٪ [CI]: ما بين 2.90–7.40)، وقيمة الـ P كانت أقل من 0.0001.

الاستنتاجات:توصلت هذه الدراسة إلى أن الملاريا المشخصة طبيًا تزيد من خطر الإصابة بالأنيميا للأطفال في سن ما قبل الدراسة. وبالتالي، يجب أن يكون القضاء على الطفيليات المسببة للملاريا من دم الأطفال في سن ما قبل الدراسة سريعًا وكاملاً لمنع تطور الملاريا غير المصحوبة بمضاعفات إلى عدوى مزمنة والتي يمكن أن تؤدي إلى حدوث فقر الدم الناتج من الإصابة بالملاريا.

Translated from English version into Arabic by Asmaa Elkordy, Revised by Amal Imam, through

TRANSLATORS
WITHOUT BORDERS

学龄前儿童中临床疟疾和贫血潜在风险的关系：基于人群的 2015–2016 年马拉维微量营养素调查研究

Peter Austin Morton Ntenda, Sosten Chilumpha, Edward Tisungane Mwenyenkulu, Jane Flora Kazambwe and Walaa El-Meidany

摘要
引言：在许多热带和亚热带地区，贫血和疟疾是学龄前儿童的常见且危及生命的疾病，马拉维也不例外。本研究旨在调查马拉维学龄前儿童临床疟疾转诊与贫血(血红蛋白[Hb]＜110 g/L)的关系。
方法：利用 2015–2016 年马拉维微量营养素调查(MNS)的横断面数据，建立多元 logistic 回归模型以满足复杂的调查设计。对 1051 例 6–59 月龄儿童的血样评估疟疾（使用快检诊断试剂[RDT]-SD BIOLINE 检测 P.f/Pan 富组氨酸蛋白抗原（HRP-II)™)、血红蛋白(使用 HemoCue 301)，α-1-酸性糖蛋白(AGP)和血清铁蛋白生物标志物(使用简单的夹心酶联免疫吸附试验法即 ELISA)指标，对干血样（DBS）利用聚合酶链式反应(PCR)检测遗传性血液疾病。诊断临床疟疾以发热和快速诊断试验(RDT)阳性为依据。
结果：在 1051 例 PSC 中，29%的患者贫血，24.4%的患者因疟疾转诊。在对已知混杂因素进行调整后，有临床疟疾转诊史的 PSC 患者出现贫血的几率显著增加([a*OR*] = 4.63, 95%置信区间[*CI*]为 2.90–7.40)，$P < 0.0001$。
结论：该研究发现临床疟疾会增加 PSC 贫血的风险。因此，应快速且彻底的从 PSC 的血液中清除导致疟疾的寄生虫，以防止简单疟疾发展成一种慢性感染，从而发生疟疾相关贫血。

Translated from English version into Chinese by Peng Song, edited by Jin Chen

Paludisme clinique et risque potentiel d’anémie chez les enfants d’âge préscolaire : une étude de population de l’Enquête sur les micronutriments du Malawi 2015-2016

Peter Austin Morton Ntenda, Sosten Chilumpha, Edward Tisungane Mwenyenkulu, Jane Flora Kazambwe et Walaa El-Meidany

Résumé
Contexte : L’anémie et le paludisme sont des maladies mortelles fréquentes chez l’enfant d’âge préscolaire dans de nombreuses régions tropicales et subtropicales. Elles n’épargnent pas le Malawi. Cette étude a donc pour objectif d’étudier le paludisme clinique associé à l’anémie (hémoglobine [Hb] < 110 g/L) chez l’enfant d’âge préscolaire au Malawi.
Méthodes : À l’aide des données transversales obtenues dans le cadre de <sur les micronutriments au Malawi (EMN) de 2015-2016, des modèles de régression logistique multivariés ont été construits à l’aide d’un géomystème pour tenir compte du plan d’enquête complexe. Des échantillons de sang de 1051 enfants âgés de 6 à 59 mois ont été évalués pour le paludisme (en utilisant un test de diagnostic rapide [SDT] - SD BIOLINE Malaria Ag *Pf* / Protéine riche en histidine (HRP- II) ™, Hb (en utilisant HemoCue 301), les biomarqueurs de la glycoprotéine acide α-1 (AGP) et de la ferritine sérique (en utilisant technique de dosage immuno-enzymatique sandwich simple, ELISA) et troubles sanguins héréditaires à partir d'échantillons de sang sec (DBS) par réaction de

polymérisation en chaîne (PCR). Le diagnostic de paludisme clinique a été établi en se basant sur la fièvre et un test diagnostic rapide positif (TDR).

Résultats: Sur les 1051 PSC analysées, 29% souffraient d'anémie, tandis que 24,4% avaient été dirigées vers l'hôpital pour cause de paludisme. Après ajustement pour tenir compte des facteurs de confusion connus, les risques de survenue d'anémie chez les patients présentant des antécédents de paludisme clinique avaient augmenté (odds ratio ajusté [a *OU*] = 4,63, intervalle de confiance de 95% [*CI*]: 2,90 à 7,40), *P* 0,0001.

Conclusions : Cette étude a démontré que le paludisme clinique augmente les risques d'anémie chez l'enfant d'âge préscolaire. Par conséquent, l'élimination du sang des parasites provoquant le paludisme des enfants d'âge préscolaire, doit être rapide et totale afin de prévenir l'évolution du paludisme simple en une infection chronique pouvant causer une anémie.

Translated from English version into French by Suzanne Assenat, Revised by Eric Ragu, through

TRANSLATORS
WITHOUT BORDERS

Клиническая малярия и потенциальный риск развития анемии среди детей дошкольного возраста: популяционное исследование "Обследования микронутриентов в Малави 2015–2016 гг."

Питер Остин, Мортон Нтенда, Состен Чилумфа, Эдвард Тисунгане Мвененкулу, Джейн Флора Казамбве и Валаа Эль-Мейдани

Резюме

Справочная информация: Анемия и малярия - это распространенные и опасные для жизни заболевания среди детей дошкольного возраста во многих тропических и субтропических районах, и Малави не является исключением. Таким образом, это исследование было направлено на изучение связи реферальной клинической малярии с анемией (гемоглобин [Hb] <110 г / л) у детей дошкольного возраста в Малави.

Методы: с использованием данных поперечного сечения, полученных в ходе обследования микронутриентов в Малави ("MNS") в 2015–2016 гг., были сконструированы многомерные модели логистической регрессии с использованием геодезической логистики для учета комплексного плана обследования. Образцы крови 1051 ребенка в возрасте 6–59 месяцев были проанализированы на наличие малярии (с использованием экспресс-теста [RDT] - SD BIOLINE Malaria Ag Pf / Pan test, богатого гистидином белка (HRP-II) ™), Hb (с использованием HemoCue 301), α-1-кислого гликопротеина (AGP) и биомаркеров

сывороточного ферритина (с использованием простой методики "сэндвич"-ферментного иммуноферментного анализа, ELISA) и наследственных заболеваний крови из образцов сухой крови (DBS) с использованием полимеризационной цепной реакции (ПЦР). Диагноз клинической малярии был установлен на основании лихорадки и положительного экспресс-диагностического теста (RDT).

Результаты: из 1051 проанализированных ДДВ, 29% страдали анемией, в то время как у 24,4% имелось направление в больницу по причине малярии. После учета известных связующих элементов, ДДВ с историей рефералной клинической малярии имели бóльшие шансы развития анемии (скорректированное отношение шансов [aOR] = 4,63, 95% доверительный интервал [CI]: 2,90–7,40), P <0,0001.

Выводы: это исследование показало, что клиническая малярия повышает риск развития анемии у ДДВ. Таким образом, выведение малярийных паразитов из крови ДДВ должно быть быстрым и полным, чтобы предотвратить развитие неосложненной малярии до хронической инфекции, которая может привести к развитию связанной с ней анемии.

Translated from English version into Russian by Maria Petrenko, revised by Alexander SominMichael Orlov, through

TRANSLATORS
WITHOUT BORDERS

El paludismo clínico y el riesgo potencial de anemia entre los niños en edad preescolar: un estudio de población de la Encuesta sobre Micronutrientes de Malawi 2015–2016

Peter Austin Morton Ntenda, Sosten Chilumpha, Edward Tisungane Mwenyenkulu, Jane Flora Kazambwe y Walaa El-Meidany

Resumen

Fundamentos: la anemia y el paludismo son enfermedades potencialmente mortales entre los niños en edad preescolar en muchas zonas tropicales y subtropicales, y Malawi no es la excepción. Consecuentemente, este estudio tuvo como objetivo examinar la asociación entre la derivación por paludismo clínico y la anemia (hemoglobina [Hb] <110 g/L) en niños en edad preescolar en Malawi.

Métodos: mediante el empleo de datos transversales obtenidos de la Encuesta sobre micronutrientes de Malawi 2015–2016 (MNS, por sus siglas en inglés), se construyeron modelos de regresión logística multivariables por medio del empleo de surveylogistic para explicar el complejo diseño de la encuesta. En muestras de sangre de 1051 niños de entre 6 y 59 meses se evaluaron el paludismo (utilizando las pruebas de diagnóstico rápido [PDR]: SD BIOLINE Malaria Ag *P.f*/Pan test

histidine-rich protein (HRP-II)™), la Hb (mediante el uso de HemoCue 301), la α 1- glycoproteína ácida (AGP) y los biomarcadores de ferritina sérica (empleando la técnica de ensayo por inmunoabsorción ligado a enzimas, ELISA, por sus siglas en inglés), y los trastornos hereditarios de la sangre en muestras de sangre seca (DBS, por sus siglas en inglés) utilizando la reacción en cadena de la polimerasa (PCR, por sus siglas en inglés). El diagnóstico de paludismo clínico se realizó sobre la base de fiebre y una prueba de diagnóstico rápido (RDT, por sus siglas en inglés) positiva.

Resultados: de los 1051 niños en edad preescolar analizados, un 29 % tenían anemia, mientras que un 24,4 % habían sido derivados al hospital debido al paludismo. Tras los ajustes de las variables de confusión conocidas, los niños en edad preescolar con una historia de derivación por paludismo clínico tenían mayores probabilidades de ser anémicos (oportunidad relativa ajustada [*OR*] = 4,63, intervalo de confianza de 95 % [*IC*]: 2,90 –7,40), $p < 0,0001$.

Conclusiones: este estudio halló que el paludismo clínico aumentaba el riesgo de anemia en los niños de en edad preescolar. Por lo tanto, la eliminación de los parásitos causantes del paludismo en la sangre del niño en edad preescolar debe ser rápida y completa, de modo de prevenir el avance desde el paludismo sin complicaciones hasta una infección crónica que pueda conducir al desarrollo de la anemia relacionada con el paludismo.

Translated from English version into Spanish by MARIA JULIA GALLES DE ROIS, Revised by Patricia Martinez, through

TRANSLATORS
WITHOUT BORDERS
